# Successful removal of a telephone cable, a foreign body through the urethra into the bladder: a case report

**DOI:** 10.1186/1752-1947-1-153

**Published:** 2007-11-27

**Authors:** Ravi K Trehan, Athar Haroon, Shaukat Memon, Derek Turner

**Affiliations:** 1 SpR, Trauma & Orthopaedics, St George's Hospital, London, UK; 2 SpR, Radiology, Pilgrim Hospital, Boston, Lincolnshire, UK; 3 Associate Specialist, Urology, Pilgrim Hospital, Boston, Lincolnshire, UK; 4 Consultant Urologist, Pilgrim Hospital, Boston, Lincolnshire, UK

## Abstract

The variety of foreign bodies inserted into or externally attached to the genitourinary tract defies imagination and includes all types of objects. The frequency of such cases renders these an important addition to the diseases of the genitourinary organs. The most common motive associated with the insertion of foreign bodies into the genitourinary tract is sexual or erotic in nature. In adults this is commonly caused by the insertion of objects used for masturbation and is frequently associated with mental health disorders. We report a case of insertion of telephone cable wire into the urethra. Our case highlights the importance of good history, clinical examination, relevant radiological investigation and simple measures to solve the problem.

## Introduction

The variety of foreign bodies inserted into or externally attached to the genitourinary tract defies imagination and includes all types of objects[1-3,5,6]. The frequency of such cases renders these an important addition to the diseases of the genitourinary organs [1,2]. The most common motive associated with the insertion of foreign bodies into the genitourinary tract is sexual or erotic in nature[2]. In adults this is commonly caused by the insertion of objects used for masturbation and is frequently associated with mental health disorders [3].

## Case Presentation

**Figure 1:**
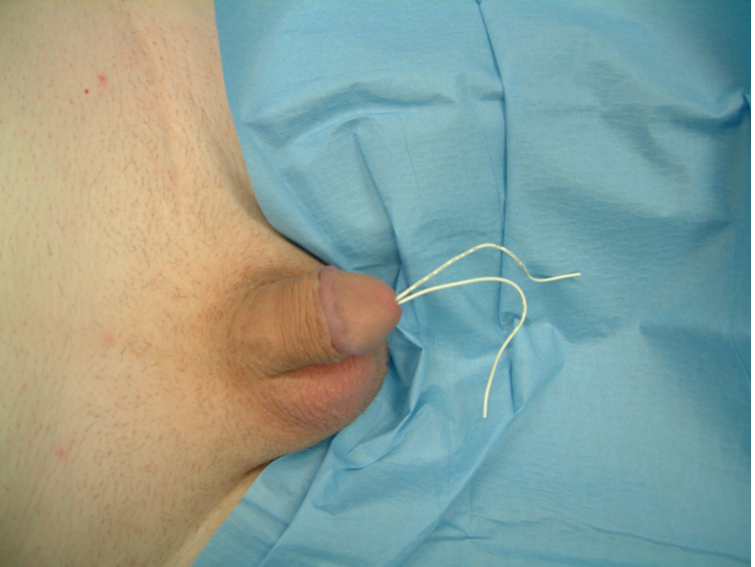
Foreign body as shown.

**Figure 2:**
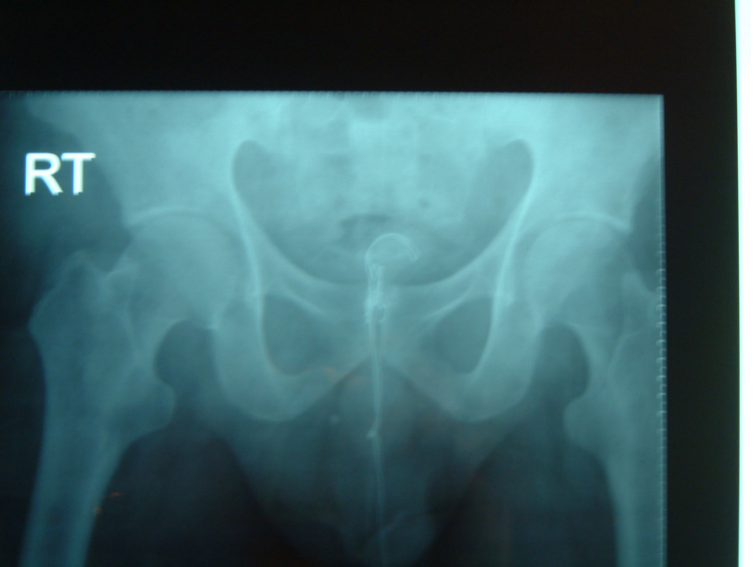
X-ray showing foreign body deep in bladder.

**Figure 3:**
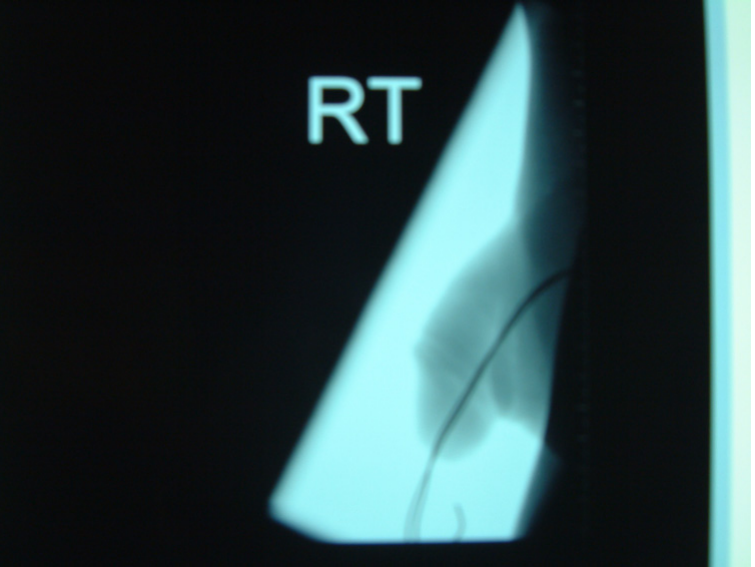
Lateral view showing foreign body.

**Figure 4:**
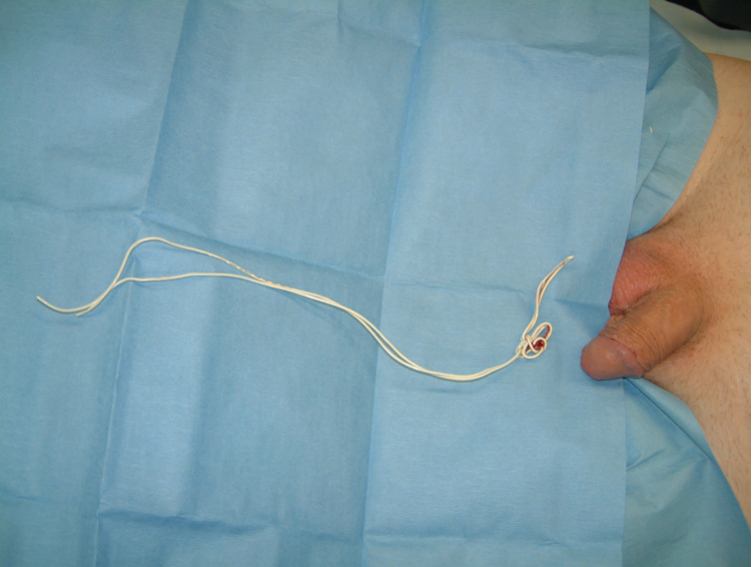
Telephone wire after successful removal.

A fifty-year-old man presented with history of urethral bleeding and pain in the urethra and supra-pubic region for a few hours following insertion of a telephone wire in his urethra. He had a past history of myocardial infarction four years earlier, after which he lost his erections. He did not opt for any treatment for his impotence. The patient gained sexual gratification after inserting a thin telephone cable wire into his urethra. He had been doing this for the last three years to get erections and after masturbation he would pull the wire out. This time after repeating the same act, he was unable to pull the wire out. He tried to pull hard but this was followed by bleeding from the urethra and soon he became incontinent. Examination revealed a thin telephone wire with two ends protruding about 5 inches out of the penis (Fig 1). The patient was incontinent and dribbling urine with spasmodic pain in the supra-pubic region. Initial attempts in the emergency department to remove the foreign body failed at which point the urology team at the hospital was involved. X-ray advised by us (Fig 2, 3) revealed a, smooth and coiled wire in the urethra and urinary bladder. Plenty of local anaesthetic gel was used and the wire was pulled out with some difficulty (Fig 4). This procedure in the Emergency Department was performed under local anaesthetic only without any sedation and with a single adult dose of intravenous gentamicin. After the patient passed urine normally, he was discharged with an appointment for follow up cystoscopy but failed to attend. His general practitioner was informed about this episode and advice for psychiatric referral was given.

## Discussion

The presence of a foreign body in the genitourinary tract represents a urologic challenge that often requires prompt intervention [1,2,4]. The most suitable method of removing any urethral foreign body depends on the size and mobility of the object in the genitourinary tract [1,2,4]. Numerous cases of intra-urethral foreign bodies of great variety and unusual nature have been reported [1-3,5,6]. Such foreign bodies are usually introduced for sexual stimulation and/or during an intoxicated or confused state. Resulting symptoms usually involve urinary frequency, dysuria, nocturia, hematuria, gross bleeding from the urethra, difficulty in voiding, or complete urinary retention[1,2].

Once a good history has been taken, detecting and investigating a possible foreign body should be done by x-ray or ultrasonography[2,8] or rarely by CT scan. Intravenous or retrograde urography may contribute additional information particularly in the case of a foreign body in the proximal genitourinary tract. Depending on the type of foreign body and its location, various methods of removal have been described, including meatotomy, cystoscopy, internal or external urethrotomy, suprapubic cystotomy, Fogarty catheterization, and injection of solvents. Endoscopic removal of these foreign bodies is often considered the treatment of choice. One may require grasping instruments including forceps, stone retrieval baskets, snares and other modified instruments[1]. The most frequent complications of foreign bodies are urethritis, urethral tear with periurethral abscess and or fistula, haemorrhage, and urethral diverticuli [7]. An early and immediate suitable treatment is recommended. It is suggested that a psychiatric evaluation should be recommended in order to discover any underlying mental health disorders, thus reducing the risk of recurrence[5].

Rahman et al[1] reported their 17 years experience with self-inflicted male urethral foreign body insertion. In all 17 patients foreign bodies were palpable. The most common symptom was frequency with dysuria. A psychiatric disorder was the most important cause, followed by intoxication and erotic stimulation. All patients had diagnostic imaging. Plain radiographs were sufficient in 14 patients, ultrasonography and CT scan was required in 3 patients. Endoscopic retrieval was successful in all but one patient. They concluded that radiological evaluation is necessary to determine the exact size, location and number of foreign bodies.

Van Ophoven et al[2] did an extensive search of the literature and revealed the results in a review article. They reviewed the literature published between 1755 and 1999. They concluded that the most common cause of foreign body insertion is sexual or erotic in nature. The most suitable method of removing a urethral foreign body depends on the size and mobility of the object. They suggested that when possible, endoscopic or minimally invasive techniques of removal should be used. In case of severe associated inflammation, surgical retrieval may be required.

In our case, with the help of X-ray we confirmed that although foreign body was inserted as far as the urinary bladder and knotted inside, it was smooth with no metal wires sticking out. We successfully removed the foreign body without the need for any surgical intervention.

## Conclusion

Removal of foreign bodies of the urogenital system should follow rules of basic surgical practice. Underlying psychiatric illness may be present and a high index of suspicion is required in the management of such patients. A plain pelvic radiograph is recommended to fully delineate all foreign bodies present.

## Competing interests

The author(s) declare that they have no competing interests.

## Authors' contributions

RT was involved in the case directly, performed the literature search and helped draft part of the manuscript.

AH was involved in the literature review and drafting of the manuscript.

SM was involved directly in the treatment of the patient and assisted in the preparation of the manuscript.

DTLT Turner was involved in overall supervision.

## Consent

The patient's informed written consent has been obtained for publication of this manuscript.
